# Pain after emergency treatments of symptomatic irreversible pulpitis and symptomatic apical periodontitis in the permanent dentition: a systematic review of randomized clinical trials

**DOI:** 10.3389/froh.2023.1147884

**Published:** 2023-10-18

**Authors:** Abdelrahman M. Alhilou, Essam Ahmed Al-Moraissi, Abdulaziz Bakhsh, Nikolaos Christidis, Peggy Näsman

**Affiliations:** ^1^ Department of Restorative Dentistry, College of Dentistry, Umm Al-Qura University, Mecca, Saudi Arabia; ^2^ Department of Oral and Maxillofacial Surgery, Faculty of Dentistry, Thamar University, Thamar, Yemen; ^3^ Division of Oral Diagnostics and Rehabilitation, Department of Dental Medicine, Karolinska Institutet, and Scandinavian Center for Orofacial Neurosciences, Huddinge, Sweden; ^4^ Division of Oral Diseases, Department of Dental Medicine, Karolinska Institutet, Huddinge, Sweden

**Keywords:** symptomatic, irreversible pulpitis, apical periodontitis, pulpectomy, pulpotomy, pain, emergency treatment

## Abstract

**Background:**

Symptomatic irreversible pulpitis (SIP) or symptomatic apical periodontitis (SAP) are two painful conditions often warranting emergency treatment. The most common emergency treatments supported by evidence are pulpotomy and pulpectomy and are normally performed under time-constrained circumstances. However, there is no strong evidence of which treatment suggested in literature a clinician can use to reduce endodontic pain effectively. Therefore, the aim of this systematic review is to investigate the present knowledge on postoperative pain related to the two types of emergency treatments available for treating SIP and SAP.

**Methods:**

Randomized controlled trials investigating postoperative pain after emergency treatments (pulpotomy and/or pulpectomy) on permanent dentition with signs and symptoms of SIP and/or SAP were searched in three major databases from 1978 until 2022. Risk of bias was assessed with Cochrane's tool.

**Results:**

Only five studies fulfilled the inclusion criteria. The included studies indicated that pulpotomy and pulpectomy are both suitable treatment options for SAP and SIP, as they provide sufficient alleviation of pain in permanent dentition. However, inconsistent results were found between the included trials on which emergency treatment is more effective in reducing pain. Cochrane's tool revealed that the studies had a low risk of bias. Limitations found in the design of the included randomized control trials decreased the level of evidence. None of the included studies accounted for essential confounding variables, such as factors affecting pain (including the psychological aspects). Moreover, possible non-odontogenic pain was not assessed, and therefore, it was not excluded; hence, affecting the internal validity of the studies.

**Conclusion:**

There are controversies within the available randomized control trials on which treatment is most effective in reducing emergency pain. This could be due to some weaknesses in the design of the clinical trials. Thus, further well-designed studies are warranted to draw conclusions on which emergency treatment is more effective in reducing pain.

**Systematic Review Registration:**

PROSPERO (CRD42023422282).

## 1. Introduction

Oro-facial pain, is a pain related to the mouth and/or face (1). The prevalence of orofacial pain varies between countries, i.e., 5%–57% (2); specifically, toothache or dental pain prevalence range globally between 7% and 32%, which has a psychological, social and economic negative impact on the person affected as well as on the society (3). Most of the dental pain is due to periapical or pulpal disease, requiring emergency intervention, such as endodontic treatment or extraction (4). The prevalence of pain requiring endodontic procedures is 81% (5). Endodontic emergency treatment is generally a procedure needed to soothe patients' acute symptoms and is completed in limited time, as the treatment is normally booked in emergency slots. The primary goal of the treatment is to eliminate the patient's symptoms during this short period of time. However, the goal is not always easy to achieve, especially that several factors can contribute to such pain, including the emotional experiences of the person affected and the ability of the dentist to diagnose and choose the effective treatment modality.

Pain is defined by the International Association for the Study of Pain (IASP) as “an unpleasant sensory and emotional experience associated with or resembling that associated with, actual or potential tissue damage” (6). This unpleasant subjective sensation is also related to emotional experiences. Highly anxious patients have expectations of higher pain during the treatment compared to those with a lower level of anxiety (7). Therefore, the emotional state is as important as the patients' physical symptoms and must be considered during treatment. However, it is unknown if the literature related to dental pain emergency treatments considers such aspects when evaluating pre-/post-operative pain.

Symptomatic pain that requires endodontic intervention is due to pulpal damage caused by carious lesions, cracks, trauma and/or crown preparation. When such damage occurs, the pulp starts a normal defensive mechanism and develops pulpitis (pulp inflammation) (8). Based on the clinical signs and symptoms, as well as the degree to which the pulp can heal itself, diagnosis of pulpal inflammation can be either reversible or irreversible pulpitis (9). However, irreversible pulpitis as a diagnosis is being questioned due to new evidence showing that this clinical diagnosis does not necessarily match the histologic condition of the pulp. A histological study showed that in some cases diagnosed with irreversible pulpitis, only the upper part of the pulp, which is close to caries, had bacterial invasion, while the rest of the pulp remained free of inflammation (10). Hence, if the inflamed coronal part of the pulp is removed (partial pulpotomy or pulpotomy), it will have the ability to regenerate and return to its normal state (11). However, if bacteria succeed to invade the whole pulp; as a result, the pulp becomes partially or fully necrotic (infected), which could create either symptomatic or asymptomatic apical periodontitis (8, 12, 13). Symptomatic apical periodontitis was defined as “inflammation usually of the apical periodontium, producing clinical symptoms including a painful response to biting and/or percussion or palpation. It might or might not be associated with an apical radiolucent area” (9).

Pulpotomy and pulpectomy as emergency treatments have been suggested to reduce endodontic pain (14, 15). The procedure where the infected/inflamed/necrotic pulp tissue is removed from the root canals where the pulp tissue is replaced with a root filling material is called pulpectomy. This procedure is considered a time-consuming and invasive procedure (16); however, it has a success rate of almost 90% (17). On the other hand, a pulpotomy is a procedure that can be completed in a limited time where diseased pulp tissue is excised from the pulp chamber, leaving healthy tissue intact (18). Historically, emergency pulpotomy treatment aims were first to relieve pain and second to allow the development of uncompleted root apices followed by pulpectomy after complete root formation (19). Moreover, the success rate of the treatment was considered relatively low, i.e., from 13 to 37% (20, 21). The concept of vital pulp therapy, including pulpotomy have recently changed especially with the relatively high success rates reported (11, 22). However, no firm evidence, such as a systematic review, proves the superiority of one treatment over another in reducing emergency endodontic pain. Therefore, this systematic review aims first to investigate pulpotomy as an emergency treatment of symptomatic apical periodontitis (SAP) and symptomatic irreversible pulpitis (SIP) compared to pulpectomy on postoperative pain. Second, to investigate if the psychological aspects of the patients are considered in investigated randomized control trials (RCT). Current study hypothesized that pulpotomy as an emergency treatment has less postoperative pain than pulpectomy. Moreover, no studies have investigated the psychological aspects of the patients before assessing pain.

## 2. Materials and methods

**Figure 1:**
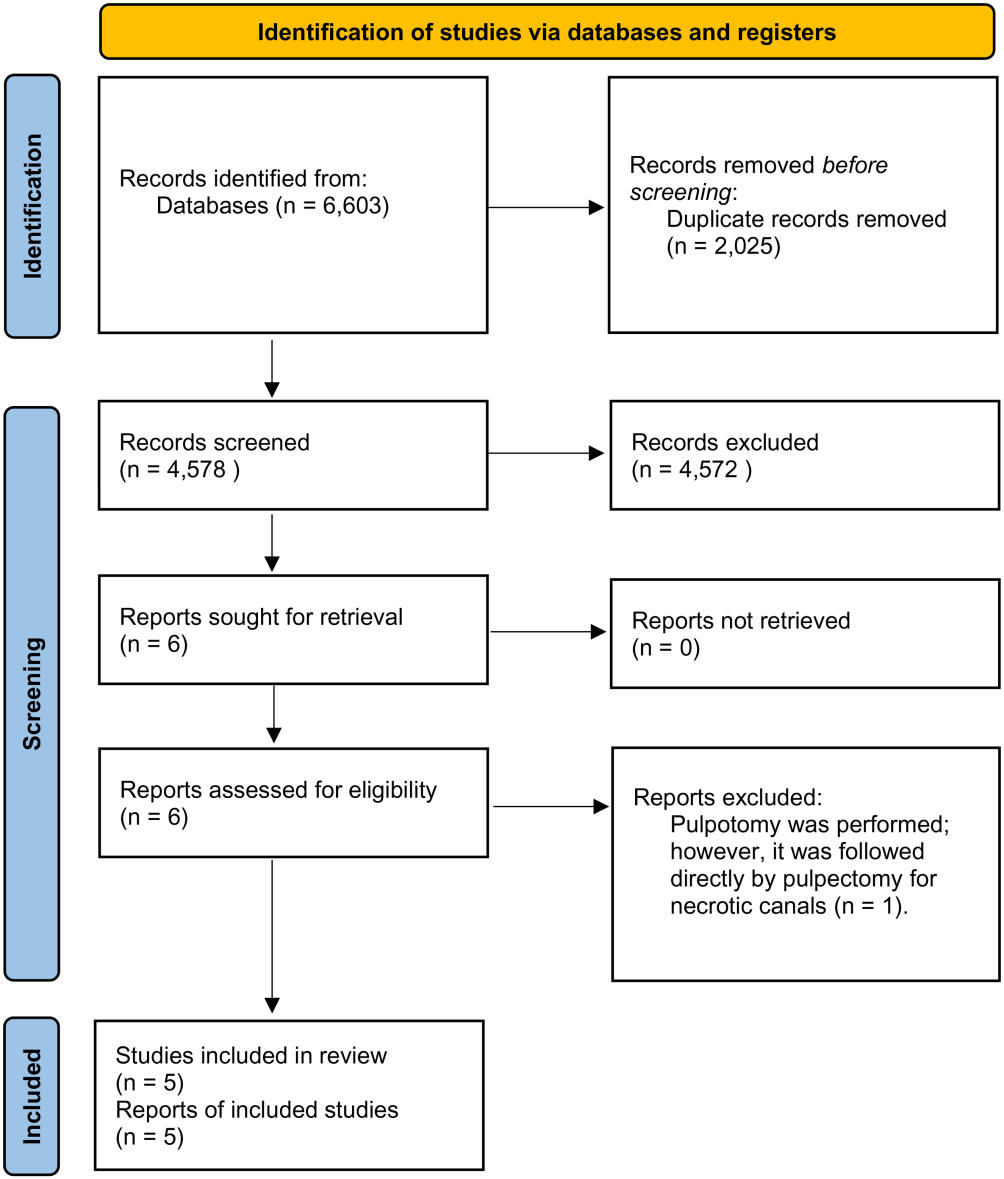
The process of identifying studies through databases and registers.

This review was done based on Preferred Reporting Items for Systematic reviews and Meta Analyses (PRISMA) statement for reporting systematic reviews (23) (Figure 1). The review was registered in PROSPERO (CRD42023422282).

### 2.1. Search strategy

The electronic databases “Medline (Ovid)”, “Embase (embase.com)” and “Web of science (Clarivate Analytics)” were used to conduct a search of clinical controlled trials published from 1978 until 2022, which investigated the use of emergency pulpotomy and/or pulpectomy to alleviate pain (toothache). The search terms and the search strategy are presented in Supplementary Material (Table 1).

**Table 1:** The table presents the population characteristics, treatment types and mean duration of treatment for the five studies included in the systematic review.

Authors, years	Mean age of participants	Treatment type	Mean duration	Male/female ratio:	Number of participants	Number of participants that did not receive allocated treatment
Eghbal et al. (24)	28 years old	(1) Pulpectomy (2) MTA pulpotomy (3) CEM pulpotomy	69.73 min 35.37 min 33.62 min	55/113 76/112 63/131	168 188 194	0 0 0
Wolf et al. (25)	46 years old	(1) Pulpectomy (2) Pulpotomy	NA NA	19/11 13/14	30 27	5 2
Eren et al. (26)	35 years old	(1) Pulpectomy (2) Partial pulpectomy (3) Pulpotomy	24 min 13 min 5 min	10/12 10/12 8/14	22 22 22	0 0 0
Galani et al. (27)	22 years old	(1) Pulpectomy (2) Pulpotomy	NA NA	11/13 15/11	27 27	0 0
Asgary and Eghbal (28)	26 years old	(1) One-visit root canal treatment (2) Pulpotomy	NA NA	82/120 72/133	202 205	0 0

NA, not applicable.

### 2.2. Study selection criteria

The PICOTS process was used to adopt the following inclusion criteria: (a) Randomized clinical trials (b) patients suffered from SIP or SAP (c) treatment performed on permanent teeth (d) complete pulpotomy or partial pulpotomy as an emergency treatment using pulpectomy as a comparative control (d) all reported follow-up times, especially for postoperative pain after emergency treatment and finally (e) pain relief. The articles were excluded if: (a) Treatment was performed on anything other than acute symptomatic tooth (b) treatment other than pulpectomy or pulpotomy (c) treatment performed on primary teeth (d) studies other than randomized control trials. The assessment for the eligibility for inclusion was done independently by 2 of the authors (AB and AA). Then, the authors conducted two rounds of meetings together and solved the heterogeneity by consensus. Therefore, the kappa statistics agreement was deemed not necessary.

### 2.3. Quality assessment

The modern version of Cochranes tool was used to evaluate risk of bias by assessing randomization, blinding of participants, blinding of assessors, drop out, reporting bias and judgement (29). Several systems can be used to classify evidence. The grading system GRADE, which stands for Grading of Recommendations Assessment, Development and Evaluation, to assess the treatment studies from strong (number 4) to weak (number 1) was first planned to use (30). However, the studies included in this review were insufficient for a meta-analysis; hence this grading system was not applicable.

## 3. Results

### 3.1. Literature search outcome

The outcome of the search from the three electronic databases gave us a result of 6,603 articles; however, 2,025 duplicate records were removed. Based on the articles' title and abstract, only six randomized control trials met the inclusion criteria (24–28, 31). However, one of these papers was excluded after reassessing the eligibility by reading the papers thoroughly (31). The reason behind the exclusion of this paper is that a pulpectomy was performed on necrotic canals for the teeth assigned for the group of pulpotomy procedure. i.e., only vital or partially vital canals received a pulpotomy procedure. Therefore, 4,573 articles were excluded (Figure 1).

### 3.2. Characteristics of included studies

The population characteristics for the studies included are presented in Table 1. Moreover, studies characteristics are presented in Table 2. Well-designed RCT is considered the most reliable evidence as it reduces the effect of confounding factors (32). Limitations are found in the design of all the included studies, i.e., Some essential factors that could affect pain evaluation were not considered (patient's psychological state, patient history of pain, the type of tooth treated, age of participants and sex); moreover, possible non-odontogenic pain was not assessed, minimizing the reliability of all included randomized control trials. None of the studies considered the psychological aspect when evaluating pre-or postoperative pain. Only one study evaluated tooth survival and the follow-up was 18 months. The study found no significant differences in tooth survival between pulpotomy and pulpectomy. The reported success rate was 85% for pulpotomy and 87% for pulpectomy (27). Only five articles were found, which was not sufficient for a meta-analysis. The longest follow up period was 5 years (28); however, no pain evaluation with numerical rating scale (NRS) was performed after seven days of treatment. Five years follow up on that study was for clinical and radiographic evaluation only. Pain follow up in the other four studies ranged from 5 to 7 days.

**Table 2:** The table present studies characteristics and selection criteria using PICOTS.

Authors, years	Type of study, level of evidence	Pulpal status	Periapical status	Intervention	Comparator	Outcome	Follow up of pain
Eghbal et al. (24)	RCT, 1b	Vital (exposure due to carious excavation) or SIP	Normal or SAP	Pulpotomy	Pulpectomy	NRS (0–9) within four grades: Pain-free Mild Moderate Severe	6, 12, 18, 24, 36 hours as well as day 3, 4, 5, 6 and 7 postoperative
Wolf et al. (25)	RCT, 1b	Necrotic pulp	Normal or SAP	Pulpotomy	Pulpectomy	NRS (0–10) 0 = no pain 10 = worst pain	3–5 days postoperative
Eren et al. (26)	RCT, 1b	SIP	Normal or SAP	Pulpotomy	Pulpectomy	VAS (0–10) 0 = no pain 10 = unbearable pain	Day 1, 3 and 7 postoperative
Galani et al. (27)	RCT, 1b	Vital (exposure due to carious excavation)	Normal	Pulpotomy	Pulpectomy	VAS (0–10) 0 = no pain 1–3 = mild 4–6 = moderate 7–10 = severe	Day 1, 2, 3, 4, 5, 6 and 7 postoperative
Asgary and Eghbal (28)	RCT, 1b	Irreversible pulpitis	Normal	Pulpotomy	Pulpectomy	NRS (0–9) 0= No pain 1–3= Mild pain 4–6= Moderate pain 7–9= Severe pain	6, 12, 18, 24, 36, 48, 60 hours as well as day 3, 4, 5, 6, and 7 post-operative

RCT, randomized control trial; SAP, symptomatic apical periodontitis; SIP, symptomatic irreversible pulpitis; NRS, numeric rating scale; VAS, visual analogue scale.

### 3.3. Pulpectomy vs. pulpotomy as an emergency endodontic treatment

The five clinical trials which satisfied the inclusion criteria showed that pulpotomy and pulpectomy are both suitable treatment options for SIP and SAP, as they provide sufficient pain relief in permanent dentition. Studies have used different methods to evaluate postoperative pain. Two studies used the numeric rating scale for pain assessment (24, 25) found no differences between pulpotomy and pulpectomy in reducing pain up to seven days postoperatively (24, 25). Another study which used NRS for pain evaluation described pulpotomy as less painful over the assessment period (28). In addition, two studies have used the visual analogue scale (VAS) to evaluate postoperative pain (26, 27). Galani et al. reported that emergency treatment using pulpotomy was less painful than pulpectomy, in contrast, Eren et al. found the opposite, i.e., pulpectomy reduced postoperative pain more than pulpotomy.

### 3.4. Risk of bias assessment

Only one study was single-blinded (26). Application of blinding was difficult due to the nature of the research. All included studies reported risk of bias except one (25). The evidence had a low risk of bias in all included articles (Table 3).

**Table 3:** Table representing cochranes “Risk of bias”.

Authors, years	Randomisation	Allocation concealment	Blinding of participants and personal	Blinding of assessor	Drop out	Reporting bias	Judgment
Eghbal et al. (24)	YES	YES	NO	YES	NO	YES	YES
Wolf et al. (25)	YES	NO	YES	NO	YES	NO	NO
Eren et al. (26)	YES	YES	YES (only patients)	NO	NO	YES	NO
Galani et al. (27)	YES	YES	NO	NO	YES	YES	NO
Asgary and Eghbal (28)	YES	YES	YES	NO	NO	YES	NO

## 4. Discussion

The findings of the current review revealed that both pulpotomy and pulpectomy are effective emergency treatments to reduce endodontic related pain. The limitation found in the design of all the included studies reduced the quality of evidence from strong (randomized control trials), to moderate evidence.

Studies varied in tools used for pain assessment. Aforementioned, some studies that considered NRS to evaluate pain reported no differences between pulpotomy and pulpectomy in reducing endodontic pain (24, 25), while others using the same tool found a difference (28). Moreover, studies which assessed the post-operative pain through the VAS reported differences between treatments (26, 27). Although the reliability and validity of both VAS and NRS to measure pain intensity are previously supported by evidence (33, 34), studies showed a slight preference toward NRS over VAS scales. For example, it has been reported that VAS is not always normally distributed (35). Moreover, one study had shown the superiority of NRS on responsivity to pain compared to VAS (36). Nevertheless, both VAS and NRS scales are only tools to record subjective symptoms that can be affected by several factors. The patient's psychological state is one of the most important factors that affect pain (37). Highly anxious patients have higher pain expectations during endodontic treatment than those with a lower level of anxiety (7). Furthermore, pain past experiences have an impact on the threshold of individuals and can be a valuable tool for good diagnosis (7, 8). Other factors could include the type of tooth treated (multi-rooted or single-rooted teeth), age of participants and sex (38). According to epidemiological studies, females have a higher prevalence of chronic pain than males (39). Evidence shows that females are more prone to temporomandibular disorder (a chronic pain affecting orofacial region) than males (40, 41). Unfortunately, none of the five randomized control trials included in the current review considered the psychosocial state of participants when comparing treatments. Only one study had a past history of pain in their inclusion criteria for diagnosis of irreversible pulpitis; however, it was not considered when pain intensity was analysed between treatments (24). All the studies used a single type of tooth (standard methodology), except for one study. In that study, the pre-/post-operative pain was reported for each treatment on each tooth type. However, the pain intensity reported for each tooth type was not compared between treatments (25). In all of the studies, age and sex data were reported but was not used for further analysis when pain intensity was compared between treatments. All previously mentioned factors could be considered confounding factors; and most probably this is why results differ between these clinical trials. These confounding factors are limitations in the design of the studies, hence reducing their quality.

Pain caused by a physical issue can be explained and treated easily: however, pain that is unusual or has no apparent cause can be more confusing and frustrating. Referred pain and psychosomatic pain are other significant problems. Psychosomatic pain is when psyche issues such as depression, anxiety, or stress induce pain that spreads to other body parts (42). On the other hand, referred pain is when the patient feels symptoms in a different location from where the cause of the pain is. Referred pain and psychosomatic pain leads to difficulty in identifying the source of patients' symptoms. The origin of intradental pain could be odontogenic, non-odontogenic or even systematic. Referred pain from the masticatory muscles, suboccipital muscles or the temporomandibular joints are examples of non-odontogenic pain often misdiagnosed with odontogenic pain. However, referred pain from the central nervous system (systematic) such as trigeminal neuralgia or atypical facial neuralgia can also lead to a missed diagnosis (8). No studies included diagnostic tests rather than clinical dental diagnosis, including pulp sensitivity tests that have limitations (43), i.e., studies show a poor correlation between pulp sensitivity tests and the histological state of the pulp (44, 45). Hence, the internal validity of the trials is mostly affected. Therefore, one can suggest the diagnostic criteria of temporomandibular joint disorder (TMD) (DC/TMD) (46) as an additive value for future research concerning the current topic to minimise the possibilities of misdiagnosis (comorbidity). DC/TMD Axis I is a valid diagnostic tool for detecting and differentiating TMD related pain (sensitivity ≥0.86, specificity ≥0.98). This will help researchers to exclude non-odontogenic pain before conducting trials. Moreover, the DC/TMD Axis II questionnaire will help assess the participants' behavioural and psychosocial status and correlate it with pre-/post-operative pain intensity. Hence, it will reduce the possibility of having psychosocial confounding factor (previously mentioned in the first paragraph) on future study designs.

Each emergency treatment has its pros and cons. Pulpotomy is a treatment in which the pulp tissue of the pulp chamber is removed. The dentist does not penetrate the canal(s) of the tooth, and the patient is recommended to return for a complete root canal treatment. Some studies suggested that pulpotomy does not require the use of rubber dam, nor the change of instrumentation to sterile ones, as the canals are not penetrated (14, 47). This saves time during emergency treatment, and time is critical in an emergency visit. Nonetheless, recent studies are emphasizing the importance of using rubber dam during pulpotomy which will improve the success rate of the treatment, especially in cases diagnosed with reversible or irreversible pulpitis (11, 22). Pulpectomy starts the same way as a pulpotomy; however, it is continued by chemo-mechanical treatment of the root canal system by application of rubber dam to prevent any contamination of the area. Pulpectomy is a more time-consuming treatment than pulpotomy, but the success rate reported in the literature is higher than pulpotomy (16). The most probable reasons for pain relief in both emergency treatments are intra-pulpal reduction of tissue pressure and the concentration of inflammatory mediators. Moreover, excision of the inflamed coronal part of the pulp or the whole pulp will reduce or eliminate the number of nociceptive sensory free nerve endings (48). However, there is still a significant gap in the literature that has to be filled related to which emergency treatment is most effective in reducing pain.

## 5. Conclusion

Within the few articles found, the results of this systematic review show that both pulpectomy and pulpotomy as emergency treatments can reduce pain in permanent dentition with signs and symptoms of SIP and/or SAP. However, there are still controversies within these moderate-quality papers on which treatment is most effective in reducing pain. The controversy could be due to cofounding factors not considered during pain evaluation in any of the investigated randomized control trials. Hence, there is still a big need for better designed randomized control trials assessing the best possible emergency treatment option in minimizing the pain.

## Data Availability

The original contributions presented in the study are included in the article/**Supplementary Material**, further inquiries can be directed to the corresponding author.
