# Supplementary material for: Pain after emergency treatments of symptomatic irreversible pulpitis and symptomatic apical periodontitis in the permanent dentition: a systematic review of randomized clinical trials
Source: Front Oral Health. 2023 Oct 18;4:1147884. doi: 10.3389/froh.2023.1147884 (PMC10618681; doi:10.3389/froh.2023.1147884)
Supplement: Supplementary file 1 [file Table1.docx]

Supplementary Material

Pain after emergency treatments of symptomatic irreversible pulpitis and symptomatic apical periodontitis in the permanent dentition: a systematic review of randomized clinical trials

Abdelrahman M. Alhilou*, Essam Ahmed Al-Moraissi, Abdulaziz Bakhsh, Nikolaos Christidis, Peggy Näsman

*** Correspondence:** Abdelrahman M. Alhilou: amhilou@uqu.edu.sa

# Supplementary Figures and Tables

**Table1.** The table is showing the search strategy and mesh words used to search for randomized controlled trial literature from 1983 - 2022 in **1.** Medline, **2.** Embase and **3**. Web of science.

1. Medline

| Interface: Ovid MEDLINE(R) and Epub Ahead of Print, In-Process & Other Non-Indexed Citations and Daily  Date of Search: February 8, 2022  Number of hits: 3,049  Comment: In Ovid, two or more words are automatically searched as phrases; i.e. no quotation marks are needed | Field labels   - exp/ = exploded MeSH term - / = non exploded MeSH term - .ti,ab,kf. = title, abstract and author keywords - adjx = within x words, regardless of order - * = truncation of word for alternate endings |
| --- | --- |
| \| **#** \| **Searches** \| **Results** \| \| --- \| --- \| --- \| \| 1 \| Toothache/ \| 2864 \| \| 2 \| exp Dental Pulp Diseases/ \| 11566 \| \| 3 \| exp Periapical Diseases/ \| 7846 \| \| 4 \| ((dental or endodontic or pulp* or teeth or tooth) adj3 inflammation*).ti,ab,kf. \| 1402 \| \| 5 \| ((periapical or pulp) adj3 disease*).ti,ab,kf. \| 980 \| \| 6 \| ((dental or endodontic or pulp* or teeth or tooth) adj3 pain*).ti,ab,kf. \| 4525 \| \| 7 \| (pulp adj3 (autolys?s or calcification* or exposure or gangrene or necrosis or test)).ti,ab,kf. \| 2272 \| \| 8 \| ("anomalous dysplasia of dentin" or acute nonsuppurative periodontit* or apical alveolar abscess* or apical dentoalveolar abscess* or apical periodontit* or dental granuloma* or denticle* or dentin anomalous dysplasia* or dentin dysplasia* or devitalized teeth or devitalized tooth or endodontically-treated teeth or endodontically-treated tooth or nonvital teeth or nonvital tooth or odontalgia* or periapical abscess* or periapical granuloma* or periapical periodontit* or pulpal dysplasia* or pulp gangrene* or pulpitides or pulpitis or pulpless teeth or pulpless tooth or pulp mummification* or pulp necros?s or pulp stone* or radicular cyst* or secondary dentin* or toothache* or tooth ache*).ti,ab,kf. \| 11723 \| \| 9 \| or/1-8 \| 30400 \| \| 10 \| Pulpotomy/ \| 1644 \| \| 11 \| Pulpectomy/ \| 1177 \| \| 12 \| exp Root Canal Therapy/ \| 21985 \| \| 13 \| Endodontics/ \| 2430 \| \| 14 \| Pain Management/ \| 38667 \| \| 15 \| exp Periapical Diseases/th \| 1598 \| \| 16 \| exp Dental Pulp Diseases/th \| 3050 \| \| 17 \| Toothache/th \| 406 \| \| 18 \| (pulpectomy or pulpotomy or pulpectomies or pulpotomies).ti,ab,kf. \| 1908 \| \| 19 \| ((pulp or root canal) adj3 (manage* or obturation* or preparation* or procedure* or therap* or treat*)).ti,ab,kf. \| 10574 \| \| 20 \| (apexification* or apexogenes?s or dental pulp devitalization or retrograde obturation*).ti,ab,kf. \| 668 \| \| 21 \| (endodontic adj3 (manage* or method* or obturation* or procedure* or technique* or therap* or treat*)).ti,ab,kf. \| 8937 \| \| 22 \| ((pain or toothache* or tooth ache*) adj3 (manage* or therap* or treat*)).ti,ab,kf. \| 85877 \| \| 23 \| (emergency adj3 (manage* or therap* or treat*)).ti,ab,kf. \| 22735 \| \| 24 \| or/10-23 \| 165234 \| \| 25 \| randomized controlled trial.pt. \| 558103 \| \| 26 \| controlled clinical trial.pt. \| 94685 \| \| 27 \| randomi?ed.ab. \| 657456 \| \| 28 \| placebo.ab. \| 225448 \| \| 29 \| drug therapy.fs. \| 2440019 \| \| 30 \| randomly.ab. \| 375616 \| \| 31 \| trial.ab. \| 586711 \| \| 32 \| groups.ab. \| 2308447 \| \| 33 \| 25 or 26 or 27 or 28 or 29 or 30 or 31 or 32 \| 5272871 \| \| 34 \| exp animals/ not humans.sh. \| 4955300 \| \| 35 \| 33 not 34 \| 4589970 \| \| 36 \| 9 and 24 and 35 \| 3049 \| | |

2. Embase

| Interface: embase.com  Date of Search: February 8, 2022  Number of hits: 2,673  Comment: Emtree is the controlled vocabulary in Embase | Field labels   - /exp = exploded Emtree term - /de = non exploded Emtree term - ti,ab,kw = title, abstract and author keywords - NEAR/x = within x words, regardless of order - * = truncation of word for alternate endings |
| --- | --- |
| **No.**  **Query**  **Results**  **2,673**  **#28**  **#11** AND **#26** AND **#27**  **4,502,153**  **#27**  (**'randomized controlled trial'**/de OR **'controlled clinical trial'**/de OR **random?**:ti,ab OR **'randomization'**/de OR **'intermethod comparison'**/de OR **placebo**:ti,ab OR **compare**:ti OR **compared**:ti OR **comparison**:ti OR ((**evaluated**:ab OR **evaluate**:ab OR **evaluating**:ab OR **assessed**:ab OR **assess**:ab) AND (**compare**:ab OR **compared**:ab OR **comparing**:ab OR **comparison**:ab)) OR ((**open** NEXT/1 **label**):ti,ab) OR (((**double** OR **single** OR **doubly** OR **singly**) NEXT/1 (**blind** OR **blinded** OR **blindly**)):ti,ab) OR **'double blind procedure'**/de OR **'parallel group?1'**:ti,ab OR **crossover**:ti,ab OR **'cross over'**:ti,ab OR (((**assign?** OR **match** OR **matched** OR **allocation**) NEAR/5 (**alternate** OR **group?1** OR **intervention?1** OR **patient?1** OR **subject?1** OR **participant?1**)):ti,ab) OR **assigned**:ti,ab OR **allocated**:ti,ab OR ((**controlled** NEAR/7 (**study** OR **design** OR **trial**)):ti,ab) OR **volunteer**:ti,ab OR **volunteers**:ti,ab OR **'human experiment'**/de OR **trial**:ti) NOT (((**random?** NEXT/1 **sampl?** NEAR/7 (**'cross section?'** OR **questionnaire?1** OR **survey?** OR **database?1**)):ti,ab) NOT (**'comparative study'**/de OR **'controlled study'**/de OR "randomi$ed controlled":ti,ab OR **'randomly assigned'**:ti,ab) OR (**'cross-sectional study'**/de NOT (**'randomized controlled trial'**/de OR **'controlled clinical study'**/de OR **'controlled study'**/de OR "randomi$ed controlled":ti,ab OR **'control group?1'**:ti,ab)) OR (((**case** NEXT/1 **control?**):ti,ab) AND **random?**:ti,ab NOT "randomi$ed controlled":ti,ab) OR (**'systematic review'**:ti NOT (**trial**:ti OR **study**:ti)) OR (**nonrandom?**:ti,ab NOT **random?**:ti,ab) OR **'random field?'**:ti,ab OR ((**'random cluster'** NEAR/3 **sampl?**):ti,ab) OR (**review**:ab AND **term**:it NOT **trial**:ti) OR (**'we searched'**:ab AND (**review**:ti OR **term**:it)) OR **'update review'**:ab OR ((**databases** NEAR/4 **searched**):ab) OR ((**rat**:ti OR **rats**:ti OR **mouse**:ti OR **mice**:ti OR **swine**:ti OR **porcine**:ti OR **murine**:ti OR **sheep**:ti OR **lambs**:ti OR **pigs**:ti OR **piglets**:ti OR **rabbit**:ti OR **rabbits**:ti OR **cat**:ti OR **cats**:ti OR **dog**:ti OR **dogs**:ti OR **cattle**:ti OR **bovine**:ti OR **monkey**:ti OR **monkeys**:ti OR **trout**:ti OR **marmoset?1**:ti) AND **'animal experiment'**/de) OR (**'animal experiment'**/de NOT (**'human experiment'**/de OR **'human'**/de)))  **297,629**  **#26**  **#12** OR **#13** OR **#14** OR **#15** OR **#16** OR **#17** OR **#18** OR **#19** OR **#20** OR **#21** OR **#22** OR **#23** OR **#24** OR **#25**  **31,493**  **#25**  (**emergency** NEAR/3 (**manage*** OR **therap*** OR **treat***)):ti,ab,kw  **127,475**  **#24**  ((**pain** OR **toothache*** OR **'tooth ache*'**) NEAR/3 (**manage*** OR **therap*** OR **treat***)):ti,ab,kw  **8,829**  **#23**  (**endodontic** NEAR/3 (**manage*** OR **method*** OR **obturation*** OR **procedure*** OR **technique*** OR **therap*** OR **treat***)):ti,ab,kw  **609**  **#22**  **apexification***:ti,ab,kw OR apexogenes$s:ti,ab,kw OR **'dental pulp devitalization'**:ti,ab,kw OR **'retrograde obturation*'**:ti,ab,kw  **10,063**  **#21**  ((**pulp** OR **'root canal'**) NEAR/3 (**manage*** OR **obturation*** OR **preparation*** OR **procedure*** OR **therap*** OR **treat***)):ti,ab,kw  **1,868**  **#20**  **pulpectomy**:ti,ab,kw OR **pulpotomy**:ti,ab,kw OR **pulpectomies**:ti,ab,kw OR **pulpotomies**:ti,ab,kw  **10**  **#19**  **'periodontal cyst'**/dm_th  **655**  **#18**  **'pulpitis'**/dm_th  **1,461**  **#17**  **'tooth periapical disease'**/dm_th  **2,155**  **#16**  **'tooth pulp disease'**/dm_th  **615**  **#15**  **'tooth pain'**/dm_th  **140,265**  **#14**  **'analgesia'**/de  **28,859**  **#13**  **'endodontics'**/de  **6,186**  **#12**  **'endodontic procedure'**/exp  **34,444**  **#11**  **#1** OR **#2** OR **#3** OR **#4** OR **#5** OR **#6** OR **#7** OR **#8** OR **#9** OR **#10**  **12,025**  **#10**  **'anomalous dysplasia of dentin'**:ti,ab,kw OR **'acute nonsuppurative periodontit*'**:ti,ab,kw OR **'apical alveolar abscess*'**:ti,ab,kw OR **'apical dentoalveolar abscess*'**:ti,ab,kw OR **'apical periodontit*'**:ti,ab,kw OR **'dental granuloma*'**:ti,ab,kw OR **denticle***:ti,ab,kw OR **'dentin anomalous dysplasia*'**:ti,ab,kw OR **'dentin dysplasia*'**:ti,ab,kw OR **'devitalized teeth'**:ti,ab,kw OR **'devitalized tooth'**:ti,ab,kw OR **'endodontically-treated teeth'**:ti,ab,kw OR **'endodontically-treated tooth'**:ti,ab,kw OR **'nonvital teeth'**:ti,ab,kw OR **'nonvital tooth'**:ti,ab,kw OR **odontalgia***:ti,ab,kw OR **'periapical abscess*'**:ti,ab,kw OR **'periapical granuloma*'**:ti,ab,kw OR **'periapical periodontit*'**:ti,ab,kw OR **'pulpal dysplasia*'**:ti,ab,kw OR **'pulp gangrene*'**:ti,ab,kw OR **pulpitides**:ti,ab,kw OR **pulpitis**:ti,ab,kw OR **'pulpless teeth'**:ti,ab,kw OR **'pulpless tooth'**:ti,ab,kw OR **'pulp mummification*'**:ti,ab,kw OR "pulp necros$s":ti,ab,kw OR **'pulp stone*'**:ti,ab,kw OR **'radicular cyst*'**:ti,ab,kw OR **'secondary dentin*'**:ti,ab,kw OR **toothache***:ti,ab,kw OR **'tooth ache*'**:ti,ab,kw  **2,226**  **#9**  (**pulp** NEAR/3 (autolys$s OR **calcification*** OR **exposure** OR **gangrene** OR **necrosis** OR **test**)):ti,ab,kw  **5,195**  **#8**  ((**dental** OR **endodontic** OR **pulp*** OR **teeth** OR **tooth**) NEAR/3 **pain***):ti,ab,kw  **924**  **#7**  ((**periapical** OR **pulp**) NEAR/3 **disease***):ti,ab,kw  **1,493**  **#6**  ((**dental** OR **endodontic** OR **pulp*** OR **teeth** OR **tooth**) NEAR/3 **inflammation***):ti,ab,kw  **125**  **#5**  **'periodontal cyst'**/de  **3,609**  **#4**  **'pulpitis'**/de  **7,251**  **#3**  **'tooth periapical disease'**/de  **9,217**  **#2**  **'tooth pulp disease'**/de  **8,586**  **#1**  **'tooth pain'**/de | |

3. Web of Science Core Collection

| Interface: Clarivate Analytics  Date of Search: February 8, 2022  Number of hits: 881 | Field labels   - TS/Topic = title, abstract, author keywords and Keywords Plus - NEAR/x = within x words, regardless of order - * = truncation of word for alternate endings   Note: sometimes “quotation marks” are needed for single search terms to avoid automatic term mapping (lemmatization). |
| --- | --- |
| **#15** #1 AND #8 AND #14  \| Exact search  881  **#14** #9 OR #10 OR #11 OR #12 OR #13  \| Exact search  16,925  **#13** TS=((dental OR endodontic OR pulp* OR teeth OR tooth) NEAR/2 inflammation*)  \| Exact search  1,091  **#12** TS=((periapical OR pulp) NEAR/2 disease*)  \| Exact search  611  **#11** TS=((dental OR endodontic OR pulp* OR teeth OR tooth) NEAR/2 pain*)  \| Exact search  3,862  **#10** TS=(pulp NEAR/2 (autolys$s OR calcification* OR exposure OR gangrene OR necrosis OR test))  \| Exact search  1,781  **#9** TS=("anomalous dysplasia of dentin" OR "acute nonsuppurative periodontit*" OR "apical alveolar abscess*" OR "apical dentoalveolar abscess*" OR "apical periodontit*" OR "dental granuloma*" OR denticle* OR "dentin anomalous dysplasia*" OR "dentin dysplasia*" OR "devitalized teeth" OR "devitalized tooth" OR "endodontically-treated teeth" OR "endodontically-treated tooth" OR "nonvital teeth" OR "nonvital tooth" OR odontalgia* OR "periapical abscess*" OR "periapical granuloma*" OR "periapical periodontit*" OR "pulpal dysplasia*" OR "pulp gangrene*" OR pulpitides OR pulpitis OR "pulpless teeth" OR "pulpless tooth" OR "pulp mummification*" OR "pulp necros$s" OR "pulp stone*" OR "radicular cyst*" OR "secondary dentin*" OR toothache* OR "tooth ache*")  \| Exact search  11,720  **#8** #2 OR #3 OR #4 OR #5 OR #6 OR #7  \| Exact search  115,419  **#7** TS=(emergency NEAR/2 (manage* OR therap* OR treat*))  \| Exact search  22,727  **#6** TS=((pain OR toothache* OR "tooth ache*") NEAR/2 (manage* OR therap* OR treat*))  \| Exact search  78,400  **#5** TS=(endodontic NEAR/2 (manage* OR method* OR obturation* OR procedure* OR technique* OR therap* OR treat*))  \| Exact search  6,950  **#4** TS=(apexification* OR apexogenes$s OR "dental pulp devitalization" OR "retrograde obturation*")  \| Exact search  580  **#3** TS=((pulp OR "root canal" ) NEAR/2 (manage* OR obturation* OR preparation* OR procedure* OR therap* OR treat*))  \| Exact search  9,164  **#2** TS=(pulpectomy OR pulpotomy OR pulpectomies OR pulpotomies)  \| Exact search  1,392  **#1** TS=(randomised OR randomized OR randomisation OR randomisation OR  placebo* OR (random* AND (allocat* OR assign*)) OR (blind* AND  (single OR double OR treble OR triple)))  \| Exact search  1,270,855 | |

**
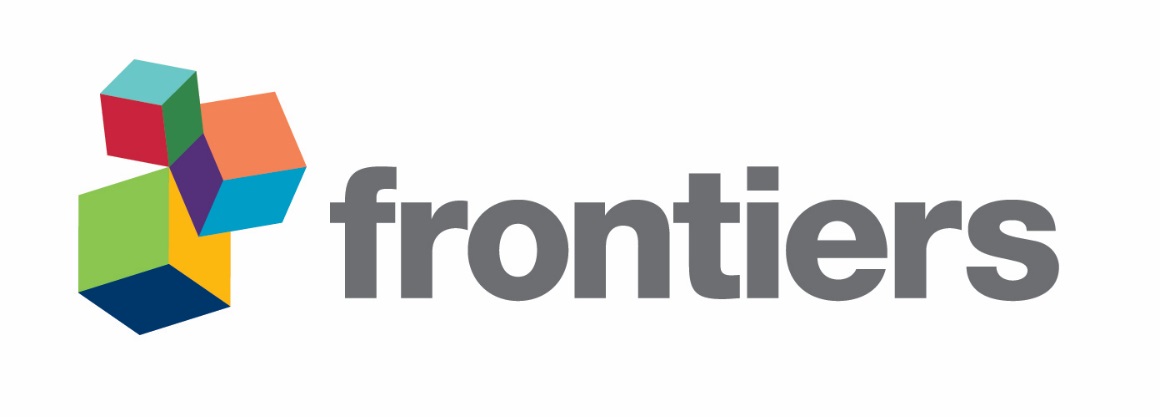
**
